# How do psychiatrists address delusions in first meetings in acute care? A qualitative study

**DOI:** 10.1186/1471-244X-14-178

**Published:** 2014-06-16

**Authors:** Alessia Zangrilli, Giuseppe Ducci, Pier Luca Bandinelli, Jemima Dooley, Rosemarie McCabe, Stefan Priebe

**Affiliations:** 1 UOC Psichiatria Ospedale San Filippo Neri, Dipartimento di Salute Mentale ASL Roma E, Via G. Martinotti 20, 00135 Roma, Italy; 2 Unit for Social and Community Psychiatry, WHO Collaborating Centre for Mental Health Services Development, Queen Mary University of London, London E13 8SP, UK

**Keywords:** Delusions, Acute care, Communication, Therapeutic relationships

## Abstract

**Background:**

Communicating about delusions can be challenging, particularly when a therapeutic relationship needs to be established in acute care. So far, no systematic research has explored how psychiatrists address patients’ delusional beliefs in first meetings in acute care. The aim of this study was to describe how psychiatrists address patients’ delusional experiences in acute in-patient care.

**Methods:**

First meetings between five psychiatrists and 14 patients in acute care were audio-recorded and analysed using thematic content analysis.

**Results:**

296 psychiatrist statements about delusions were identified and coded. Three commonly used approaches (with a total of 6 subthemes) were identified. The most common approaches were eliciting the content (1 subtheme: eliciting content and evidence) and understanding the impact (3 subthemes: identifying emotions, exploring links with dysfunctional behaviour and discussing reasons for hospital admission) while questioning the validity of the beliefs (2 subthemes: challenging content and exploring alternative explanations) was less common. The last approach sometimes put patients in a defensive position.

**Conclusions:**

Psychiatrists commonly use three approaches to address patients’ delusions in the first meeting in acute in-patient care. Questioning the patients’ beliefs can lead to disagreement which might hinder establishing a positive therapeutic relationship. Future research should explore the impact of such an approach on outcomes and specify to what extent questioning the validity of delusional beliefs is appropriate in the first meeting.

## Background

Delusions occur in patients with psychotic disorders and are frequently presented in acute situations such as hospital admissions. When patients communicate about delusions, psychiatrists have the challenging task to respond and address the experiences of patients. They need to address beliefs that they regard as false and are aware that merely arguing about the validity of the delusional content is unlikely to lead to immediate agreement. The beliefs can be seen as so far from the “norm” that they become “non-understandable” for the psychiatrist [1]. McCabe and Priebe [2] describe a commonly held view that psychiatrists should not discuss patient delusions in order to avoid inadvertently colluding with the patient’s beliefs.

A previous study using conversation analysis explored psychiatrist-patient communication about psychotic symptoms in regular out-patient consultations [3]. The findings showed that patients actively attempted to talk about the content of their delusional beliefs, while psychiatrists tended to avoid these discussions because of ensuing disagreement. The difficulties hindered engagement and led the authors to conclude that addressing delusional beliefs may be central to improving clinical communication with this patient group [2]. In psychological treatments, such as cognitive behavioural therapy, techniques have been developed to address delusions [4,5], and most research in this area has been conducted in outpatient and psychotherapy settings [3,6,7].

When psychiatrists first meet with a patient in acute in-patient care, the task of addressing delusions is even more difficult as there is no previous history with the patient and delusions are likely to be stronger than in outpatient settings. However, an appropriate response to patients’ delusions is potentially very important, as first meetings in acute treatment can strongly impact on the outcome of the treatment episode and influence long-term therapeutic relationships [8]. Patients with delusions tend to have poorer outcomes with an increased risk for rehospitalisation. Hence, there is a particular need to engage these patients in a trustful relationship from the first meeting onwards. In the first encounter already, psychiatrists are required to respond to the presentation of delusions so that patients feel understood and respected and that further treatment is facilitated [9,10]. Ideally, the response of psychiatrists should be guided by evidence about what approaches are feasible and how different responses are linked to outcomes. As a first step in such research, the range of responses used in practice needs to be assessed and understood. To date, there has been no systematic research as to how psychiatrists address delusions with patients in acute settings when they first meet them during hospital treatment. To identify different approaches, an exploratory qualitative study of first meetings between psychiatrist and patients in acute care encounters is required. The analysis of audio-taped meetings allows to study what psychiatrists really do in practice rather than what they might say in interviews about what they do.

The aim of this study was to explore different themes in how psychiatrists address patients’ delusional experiences in first routine meetings in acute in-patient care.

## Methods

### Design

This was an exploratory qualitative observational study of audio-recorded first meetings between psychiatrists and patients with delusions in routine in-patient care. Recruitment ran from April 2011 to January 2012. All ten psychiatrists from the 13-bed psychiatric in-patient service of the San Filippo Neri Hospital of Rome, Italy, were asked to participate. Five consented.

Purposive sampling was used to recruit patients from the service. Each participating psychiatrist identified recently admitted patients that fitted the inclusion criteria to approach for participation in the study.

Inclusion criteria for patients were:

•Meeting ICD-10 criteria [11] for Psychotic (F20-F29) or Mood Disorders (F30-F39)

•Presence of delusions

•Age between 18-65

•Capable of giving written informed consent.

Exclusion criteria for patients were:

•Organic impairment affecting communication.

The clinical records were used to assess whether patients met the inclusion criteria. Their suitability for the study was also discussed in routine team meetings. Following agreement amongst clinicians, psychiatrists approached eligible patients prior to the first clinical encounter, explained the study to them and obtained written informed consent.

The study adhered to RATS guidelines [12].

### Material

Consultations were audio-recorded and fully transcribed by a researcher (AZ). Patient socio-demographic and clinical data and psychiatrist socio-demographic data were documented from records.

### Analysis

The transcribed consultations were analysed using thematic content analysis [13,14]. Thematic analysis was selected specifically in order to broadly identify overarching themes in responses to patients’ communication about their delusional experiences rather than the interactional processes per se.

AZ analysed the transcripts in their entirety using a semantic approach [15]. All instances when psychiatrists addressed delusions and related experiences were identified in each interview. Each extract entailed communication in which both parties addressed delusional beliefs and related experiences. The length of extracts ranged from one brief statement or question to longer exchanges involving several statements from the psychiatrist and patient.

Each extract was coded in the first instance to characterise the psychiatrist reponse. Different themes addressing delusions were identified, each one characterized by specific verbal behaviours. The themes were then further discussed and revised in an iterative process involving all members of the interdisciplinary research team of authors which included clinical and academic psychiatrists, and clinical and academic psychologists. The findings were also repeatedly discussed in a wider research team in London (also including clinical and academic psychiatrists and psychologists as well as allied health professionals) to check and ensure internal homogeneity and external heterogeneity [16]. Following this, the themes were revised and combined into a smaller number of overarching themes.

### Ethics statement

The study has been approved by an ethics committee (Comitato Etico Lazio 1, sede Azienda Ospedaliera San Camillo Forlanni, reference: Prot. n. 605/2013 CE Lazio 1) and was compliant with the Helsinki Declaration.

## Results

### Sample

There was one female and five male psychiatrists. Their mean age was 41 years and the mean number of years working in mental health care since qualifying was 11 years.

Of the 16 patients who met the inclusion criteria during the study period and were asked to participate, 14 consented (8 female, 6 male) with a mean age of 40 years. Four of the patients were diagnosed with paranoid schizophrenia, four with other forms of schizophrenia, three with delusional disorder, two with acute psychosis, and one with depression. In nine patients, the delusions were persecutory in nature, and in the remaining patients they were hypochondriac, Cotard, thought sharing and mystic. There was no pre-existing outpatient therapeutic relationship between any of the psychiatrists and the patients in the study and for all psychiatrists, this was the first extended clinical meeting with the patient, although they may have met briefly before, during previous hospitalisations or during the current admission. Equally for the patients, this was the first extended clinical meeting during the current hospital admission, although they had reported their symptoms briefly before, e.g. during the admission process.

### Material and themes

The mean length of the recordings was 22 minutes 13 seconds, ranging from 10 minutes to 43 minutes. Across all 14 meetings, a total of 296 relevant extracts were identified in which psychiatrists addressed delusions. These extracts were grouped into six specific themes in the analysis. Each of these themes was found in at least six meetings and each psychiatrist used at least three of them. In order of frequency the themes were:

1. Eliciting the content (127 extracts)

2. Challenging content (76)

3. Exploring alternative explanations (41)

4. Identifying emotions (24)

5. Exploring links with dysfunctional behaviour (15)

6. Discussing reasons for hospital admission (13)

The distribution of specific themes across all meetings is shown in Table 1.

**Table 1:** Frequencies of psychiatrist approaches to addressing delusions across consultations

**Psychiatrist number**		**1**		**2**						**3**				**4**	**5**
**Patient number**		**1**	**6**	**2**	**3**	**4**	**5**	**13**	**14**	**9**	**10**	**11**	**12**	**7**	**8**
**Length of interview**		27:23	19:38	14:49	20:22	43:28	16:29	14:35	23:28	24:25	10:06	22:48	17:45	36:00	20:58
**Exploring content**	*Eliciting content*	✓	✓	✓	✓	✓	✓	✓	✓	✓	✓	✓	✓	✓	✓
**Understanding the impact**	*Discussing reasons for hospital admission*						✓	✓	✓	✓		✓	✓		
	*Exploring links with dysfunctional behaviour*		✓		✓		✓			✓			✓		✓
	*Identifying emotions*	✓	✓	✓		✓	✓			✓		✓			✓
**Questioning validity of beliefs**	*Challenging content*	✓	✓		✓		✓			✓		✓	✓	✓	✓
	*Exploring alternative explanations*	✓	✓		✓		✓	✓	✓	✓	✓	✓		✓	✓

These six specific themes then became subthemes when they were combined into three overarching themes: ‘Eliciting the content’ (identical with subtheme 1), ‘Understanding the impact’ (with subthemes 4, 5 and 6), and ‘Questioning the validity of the beliefs’ (with subthemes 2 and 3). Each of these overarching themes featured in at least 12 out of the 14 meetings.

### Eliciting the content and nature of the beliefs

The most frequent approach to address delusional beliefs was an attempt to elicit the content of the delusions. This was usually done in the form of simple questions. The questions aimed to understand the patient’s beliefs and encourage the patient to disclose their experience, without, however, challenging their beliefs:

PS: So, let’s say, you do not have anyone whom you can trust?

PA: No, at the end, I have realised that I cannot trust anyone…nobody….the others talk with each other….. and I am excluded, am I not?

*(patient 1, psychiatrist 1)*

PS: Where do these worms come from, can you tell me?

PA: They come from inside me.

*(patient 3, psychiatrist 3)*

PS: You always found him behind you?

PA: I always found him behind me. Of course, I now understand that I am wrong, I was worried that I would reject him and he would follow me to check my movements

PS: And that happened everyday?

PA: No, not everyday, no, every … every.. as if there were dates

PS: For example, what do you mean?

PA: As far as I know, every month, then every two months, then every three months, like this…

*(patient 4, psychiatrist 2)*

PS: Why did you feel at the centre of attention? What did you notice that gave you the impression that you were the centre of attention?

PA: Well, I felt like that for very long time, and now I understand

PS: What made you understand it?

PA: From the content of my thoughts

*(patient 5, psychiatrist 2)*

PS: That makes me think that you are saying that that theft of the motorbike did not occur by chance.

PA: … there is someone who is out to get me

*(patient 7, psychiatrist 4)*

PS: You told me even about crimes that happened during the night, about horrific situations, how can I say this, did you not

PA: in fact, we are full of blood and bruises, and talking about it with the police again…

PS: Did they confirm that situation?

PA: Yes, they said “we are all dead”

PS: also the carabinieri?

*(patient 9, psychiatrist 3)*

PS: they persecuted you, so do you want to tell me what happened?

PA: Eh… but it was all because of a group of people from a satanic sect…

*(patient 11, psychiatrist 3)*

PS: At the beginning you talked about your ability to cook

PA: Yes, I am a great chef

PS: You are a great chef?

PA: The greatest in the world

*(patient 13, psychiatrist 2)*

### Understanding the impact

#### Discussing reasons for hospital admission

When delusions (and subsequent behaviours) were reported to be the main reason for hospitalisation, psychiatrists discussed to what extent patients were aware that they had been admitted to hospital because of an illness and their delusional beliefs. Most of the time, psychiatrists used direct questions aimed at exploring the patient’s explanation for the hospitalisation or provided their own explanation:

PS: Could you please tell me why are you here? What happened?

PA: Oh, well, I called the ambulance many times for months, and the police, and firemen and the centre for victims of violence against women… cause some people followed me to trouble me or damage things

*(patient 9, psychiatrist 3)*

PS: So… what happened?

PA: Ehm… something at work, as everybody says that I bring bad luck… and when things happen it is my fault… if someone get sick it’s my fault… and everybody looks at me

*(patient 1, psychiatrist 1)*

PS: (You were admitted) in a dramatic moment, because you were very upset, worried about serious things, and ……and felt persecuted…

*(patient 11, psychiatrist 3)*

Patients responded to such questions and explanations in different ways. In some meetings, patients appreciated that the behaviour that led to hospital admission may have been inappropriate, but did not explain this through an illness or delusional beliefs:

PS: Ok, but what is the reason why you have been hospitalised?

PA: oh… is that I stripped in public…I went out naked… I left my clothes on the gate and I went to the barber shop… naked…

PS: I see…

PA: Then I went to the family doctor… still naked… and he asked me: “What are you doing?

PS: I see…

PA: And… he called the police… and sent me here…

PS: And why do think this happened, why did you undress and do all this?

PA: Because I felt like induced… forced by external factors… by hoots of cars… and I was in the spotlight… then by television, radio and even satellite…

*(patient 5, psychiatrist 2)*

Patients sometimes actively avoided talking about the symptoms leading to hospital admission, at times linked to feelings of guilt or shame. Yet, they still talked about themselves:

PS: Did the doctor tell you why he admitted you?

PA: I have understood everything. I have understood that I have pushed for it … because there, where I work….you know, I'm a very good person, I would not hurt nobody…you see that this is right…

PS: Yes, of course!

PA: I have nothing against S, or against R, or against all the people I have met here. I am from the countryside.…

*(patient 14, psychiatrist 2)*

#### Exploring links with dysfunctional behaviour

Psychiatrists tried to explore how delusions were linked with the patient’s behaviour and functioning:

PS: How did you realize that you were decomposing?

PA: Because of the strong smell

PS: Ok, but if one is decomposing, parts of the flesh should be missing, shoud they not? Because when the worms enter a dead body, I don’t know, in a forest, and nobody finds it there, for three months, the worms….

PA: I put hydrochloric acid on it, the bleach kills them

PS: Where do you put it?

PA: On those parts where they grow

PS: Did you put hydrochloric acid on your skin?

PA: Yep.

*(patient 3, psychiatrist 3)*

PS: The thought that you have to hit someone, when does it come up? ….

PA: Ehm.. it comes when the satellite influences me, sends me signals… I give you an example: I have to go to the bloke and hurt him… the satellite makes me understand that I have to do this…

*(patient 5, psychiatrist 2)*

PS: We have been told… that you do not sleep in your bed, but sometimes in a cupboard, on a chair

PA: Yes, because my bed has been broken by those people

*(patient 6, psychiatrist 1)*

#### Identifying and exploring emotions

Frequently, psychiatrists addressed emotional aspects of the experience of delusions:

PS: … But how did you feel, were you relaxed, or was there something… because several times you rang the ambulance, also the police…I assume that you were alarmed ..no?

PA: Yes, because, I saw everything. I see here too, everything in a contaminated mess

*(patient 9, psychiatrist 3)*

PS: For example, do you think it might be useful – particularly during this admission - to try and understand the emotional components which are associated with your physical sensations? That the problems are not only due to anemia, but are possibly linked to difficulties that you have emotionally and not only physically?

PA: It is both …

PS: That is one of the reasons why it is not easy to discharge you right away. So that we understand how we can help you from a physical point of view, but also from a different perspective …

*(patient 2, psychiatrist 2)*

PS: Mmm. And… Does it happen that sometimes when you feel more relaxed, less stressed, that you have doubts about this belief and maybe at times when you feel more tense, more nervous …

AP: Yes, yes, When I feel stronger, … not exhausted, then I do not think about these things

PS: And you feel more relaxed

PA: More relaxed, yes

PS: I understand. Instead, when you are under stress, this belief is stronger

PA: Exactly, yes

*(patient 5, psychiatrist 2)*

PA: But the person has no intentions to help me ... but only to make it difficult for me, with my little project

PS: Ah, I understand, and you, how did you feel like seeing all these obstacles? Were you ever angry? Were you ever

PA: Desperate

*(patient 8, psychiatrist 5)*

### Questioning the validity of the beliefs

#### Challenging the content

Psychiatrists did not just explore the content of the beliefs, but also challenged it through further questions which sometimes put patients into a position to defend their beliefs:

PS: So, they wanted to kill you and sell your organs?

PA Yes, and … sell the meat to restaurants where cannibals go…

PS: Are there restaurants for cannibals?

PA: Yes, these are secrets that the police do not know

PS: Really?

PS: This seems to be a bit difficult to believe, honestly…

*(patient 11, psychiatrist 3)*

Ps: Do you think there is any slightest chance that this is something you are exaggerating? Or that you are possibly wrong?

Pa: Nooo… I am not wrong at all.

*(patient 1, psychiatrist 1)*

PS: So… do these worms eat organs too?

PA: I think so

PS: How do you survive then, when these worms eat your organs?

PA: Well, how do I know?

*(patient 3, psychiatrist 3)*

However, in this consultation, a direct challenge makes the patient attempt to justify the belief:

PS: And if you were having thoughts, I could hear you?

PA: Yes

PS: How can this be possible? How can I hear your thoughts? I can hear only….

PA: I just don’t know, maybe because of the great burden of stress they’ve been laying on me since I was a little child

*(patient 5, psychiatrist 2)*

#### Exploring alternative explanations

Psychiatrists suggested and explored possible alternative explanations for the patients’ experiences. This was different from *challenging the content* as psychiatrists did not directly challenge the beliefs, but asked the patients only to consider the views of others or different explanations:

PS: You are describing this like a plot against you, in which at the end you were accused of having stolen a ring.

PA: Yes, but

PS: But it is not certain that this is what actually happened?

PA: But I have come to think they put me to work when I wasn’t good enough or experienced enough

*(patient 7, psychiatrist 4)*

PS: What do your parents say, given that they live with you?

*(patient 6, psychiatrist 1)*

This approach sometimes led to a defensive response of the patients too:

Ps: Did anyone tell that to your face that you bring bad luck, or is this just an interpretation you’re making of their expressions and gestures? Because there might be many reasons for example why someone can touch you…

Pa: No! Because they say “everything that has happened has been you!”

*(patient 1, psychiatrist 1)*

PS: Your parents, what do your parents for example say?

PA: They say that it is not true…

PS: Okay, they say that it is not true. And the fact that they say that it is not true, does not make you think that it is possible that it is not true, that it is perhaps rather your perception that you have worms in the body?

PA: I am decomposing

*(patient 3, psychiatrist 3)*

#### Contradictory evidence

Altogether, five more categories were identified, but featured in only four or fewer of the 14 meetings, so that they were not considered as common themes in the analysis:

1. Tracing history (a total of 10 extracts in 4 meetings)

2. Exploring links with previous stressful experiences (10/3)

3. Exploring links between discontinuing medication and symptoms (6/4)

4. Identifying coping behaviours (6/3)

5. Explaining physical symptoms as a sign of psychological distress (3/1)

## Discussion

To our knowledge, this is the first systematic qualitative analysis of how psychiatrists address delusions with acutely admitted patients. Despite high variability in the content of the delusional beliefs and the circumstances and health conditions of the participating patients, three approaches were identified that were commonly used by psychiatrists to address delusions in acute care and were used in at least 12 out of the 14 meetings. Although there was some overlap between these approaches, they were still distinct. Psychiatrists commonly asked questions to elicit the content (and sometimes evidence) of the delusional experience, explored the impact of the symptoms on the patients’ behaviour and functioning, and also questioned the validity of the beliefs by directly challenging them or offering alternative explanations. These three themes captured the common approaches that psychiatrists used to address delusional symptoms in the first extended meeting in acute treatment. Other approaches, such as exploring links with previous stressful experiences or identifying coping behaviours, were much less frequent.

McCabe et al. analysed psychiatric consultations in on-going outpatient treatment and found that psychiatrists avoided discussing delusional beliefs when they were initially raised by patients [3]. As a consequence, patients raised their beliefs again at the closing stages of the consultation, which led to explicit disagreement about the belief. In this case, psychiatrists were familiar with the patient’s beliefs and appeared to avoid discussing them as they were anticipating disagreement. The current study showed that the situation in acute care and at the first meeting appears somewhat different. Psychiatrists do not avoid talking about delusional beliefs. They address those beliefs and ask various questions to explore their content and impact.

Exploring the symptoms and finding out to what extent they influence the patient’s behaviour and functioning may be regarded as the professional duty of a psychiatrist who meets a new patient in an acute situation. In our study, all psychiatrists did this with all of their patients which might be seen as a sign of good quality of care [17]. These two approaches did not lead to any controversy with the patients and should usually be part of any first assessment in acute care.

Arguably a more difficult approach was the third one, in which psychiatrists went on to question the validity of the beliefs. In some situations, this approach put patients in a defensive position and in more or less open disagreement between patient and psychiatrist. Such disagreement arose when psychiatrists directly challenged the patients’ beliefs, but also – although less frequently – when psychiatrists suggested alternative explanations for the patients’ experience. On the one hand, such questioning might be required to check how fixed the beliefs are and distinguish them from overvalued ideas, obsessional thoughts and other phenomena that are also dysfunctional, but do not represent delusions. On the other hand, this questioning has the risk of leading to explicit disagreement in which the patient may not feel understood and respected [5]. Such disagreement might hinder the establishment of a mutually trustful relationship [18,19]. The psychiatrist has to balance two objectives in this situation, i.e. eliciting as much helpful information about the psychopathology of the patient as possible and establishing a positive therapeutic relationship as the basis for further treatment during the admission, and possibly beyond. A third objective of psychiatric consultations can be to induce therapeutic change, and questioning the patient’s beliefs is a common therapeutic approach, e.g. in cognitive behaviour therapy [20,21]. Yet, this might be less important at the first meeting during an admission than at later stages of treatment.

So, how far can and should psychiatrists go with questioning and even challenging the beliefs of patients in the first meeting? Some patients might accept and even expect to be challenged by a psychiatrist who is interested and concerned, whilst others may feel disappointed or upset by such an approach [22]. Longitudinal studies are required to provide evidence on the effect of questioning the patients’ beliefs on the therapeutic relationship, patients’ attitude to treatment and other outcomes. In the absence of such evidence, one might assume that in the first meeting, establishing a good relationship with the patient is more important than exploring all aspects of the psychopathology. The questioning can then be left for later meetings which should be easier to conduct if a good relationship has been established in the first meeting [10]. This applies in particular to the beginning of acute in-patient treatment when the patient is likely to stay in the hospital for some time and further meetings can be arranged in a flexible manner. Psychiatrists have the option of talking with patients frequently and with varying duration, depending on the preferences and response of the patient and the unfolding of the therapeutic relationship. In such a setting, initiating a useful therapeutic relationship in the first meeting may be more important than challenging delusions.

### Strengths and limitations

The study recorded a range of first meetings between psychiatrists and patients with delusional beliefs in an acute in-patient setting. It included patients with severe delusional beliefs of different kinds. Implementing such a design can be challenging, which is probably one of the reasons why – to our knowledge – it has not been done before. The analysis reached saturation in identifying core themes across almost all meetings.

There are also limitations as the study was conducted in selective samples of psychiatrists and patients in only one hospital and did not obtain data on clinical outcomes. The sample included a relatively small number of psychiatrists, with only one female.

### Implications for research and practice

Psychiatrists assessing patients in an acute setting for the first time should be aware that mere questioning of delusional beliefs – e.g. by asking for alternative explanations – can already put patients into a defensive position. If establishing a positive therapeutic relationship is a main aim of the first meeting, psychiatrists may want to be cautious when questioning the patient’s beliefs, particular when directly challenging them.

Larger studies using quantitative assessments and analyses may identify whether psychiatrists’ intervention vary depending on the subject of the delusions and to what extent other symptoms, such as current haluzinations, influence the approach of psychiatrists. Further research is required to explore in what situations such questioning – with or without subsequent disagreement – may still be appropriate and helpful, and in what situations psychiatrists should rather refrain from it. Longitudinal qualitative and quantitative studies can provide more evidence to further develop approaches for the acute situation and assess to what extent they facilitate or hinder patient engagement, shared decision making and a positive relationship.

## Conclusions

Addressing delusions presented by patients in the first meeting is a frequent challenge in acute care. Psychiatrists use mainly three distinct approaches. One of them is to question the patients’ beliefs which may lead to disagreement and jeopardise the emerging therapeutic relationship. Future research may employ experimental designs to provide evidence for which approaches are likely to lead to more or less favourable outcomes.

## Competing interests

The authors declare that they have no competing interests.

## Authors’ contributions

AZ and SP were involved in the conception and design of the study. AZ, GD and PLB participated the data collection. AZ, GD and PLC conducted the data analysis with regular input from RM and SP. AZ and SP wrote the manuscript. JD, RM approved the design and participated in the final paper. All authors read and approved the final manuscript.

## Pre-publication history

The pre-publication history for this paper can be accessed here:

http://www.biomedcentral.com/1471-244X/14/178/prepub

## References

[B1] Jaspers K General Psychopathology (Hoenig J, Hamilton M, translators) 1959 Manchester: Manchester Univerisity Press

[B2] McCabe R Priebe S Communication and psychosis: its good to talk, but how? Br J Psychiatry 2008 192 404 405 10.1192/bjp.bp.107.048678 18515888

[B3] McCabe R Heath C Burns T Priebe S Engagement of patients with psychosis in the consultation: conversation analytic study Br Med J 2002 325 1148 1151 10.1136/bmj.325.7373.1148 12433765 PMC133454

[B4] Landa Y Silverstein SM Schwartz F Savitz A Group cognitive behavioural therapy for delusions: helping patients improve reality testing J Contemp Psychother 2006 36 9 17 10.1007/s10879-005-9001-x

[B5] Freeman D Philippa G Helping patients with paranoid and suspicious thoughts: a cognitive-behavioural approach Adv Psychiatr Treat 2006 12 404 415 10.1192/apt.12.6.404

[B6] Bergmann JR Drew P, Heritage J Veiled morality: notes on discretion in psychiatry Talk at Work 1992 Cambridge: Cambridge University Press 137 162

[B7] Federico M Priebe S Fusco C Strapelli N Singh R McCabe R Communication about psychotic symptoms in long-term psychiatric care Psychopathology 2013 46 4 233 240 10.1159/000342259 23171869

[B8] Priebe S McCabe R The therapeutic relationship in psychiatric settings Acta Psychiat Scand 2006 113 69 72 10.1111/j.1600-0447.2005.00721.x 16445486

[B9] Korkeila JA Lehtinen V Tuori T Helenius H Frequently hospitalised psychiatric patients: a study of predictive factors Soc Psychiatry Psychiatr Epidemiol 1998 33 528 534 10.1007/s001270050090 9803820

[B10] Priebe S Richardson M Cooney M Adedeji O McCabe R Does the therapeutic relationship predict outcomes of psychiatric treatment in patients with psychosis? A systematic review Psychother Psychosom 2011 80 70 77 10.1159/000320976 21196804

[B11] WHO The ICD-10 Classification of Mental and Behavioural Disorders. Clinical Description and Diagnostic Guidelines 1992 Geneva: WHO

[B12] Clark JP Godlee F, Jefferson T How to peer review a qualitative manuscript Peer Review in Health Sciences 2003 2 London: BMJ Books

[B13] Boyatzis R Transforming Qualitative Information: Thematic Analysis and Code Development 1998 London: Sage

[B14] Flick U An Introduction to Qualitative Research 2002 London: Sage

[B15] Braun V Clarke V Using thematic analysis in psychology Qual Res Psychol 2006 3 77 101 10.1191/1478088706qp063oa

[B16] Patton M Qualitative Evaluation and Research Methods 1990 Newbury Park, CA: Sage

[B17] National Institute of Clinical Excellence Schizophrenia 2009 London, UK: Department of Health

[B18] Priebe S Dimic S Wildgrube C Jankovic J Cushing A McCabe R Good communication in psychiatry - a conceptual review Eur Psychiatry 2011 26 403 407 21571504 10.1016/j.eurpsy.2010.07.010

[B19] Hoaas L Lindholm SE Berge T Hagen R Hagen R, Turkington D, Berge T, Grawe R The therapeutic alliance in cognitive behavioural therapy for psychosis CBT for Psychosis: A Symptom-Based Approach 2010 London: Routledge

[B20] Kingdon D Turkington D Cognitive Therapy of Schizophrenia 2005 London New York: Guilford Press

[B21] Lewis S Tarrier N Haddock G Bentall R Kinderman P Kingdon D Siddle R Drake R Everitt J Leadley K Benn A Grazebrook K Haley C Akhtar S Davies L Palmer S Faragher B Dunn G Randomised controlled trial of cognitive-behavioural therapy in early schizophrenia: acute-phase outcomes Br J Psychiatry 2002 181 91 97 10.1192/bjp.181.43.s91 12271807

[B22] Freeman D Garety P Fowler D Kuipers E Bebbington P Dunn G Why do people with delusions fail to choose more realistic explanations for their experiences? J Consult Clin Psychol 2004 72 671 680 15301652 10.1037/0022-006X.72.4.671

